# Supplementary material for: Evidence supported by Mendelian randomization: impact on inflammatory factors in knee osteoarthritis
Source: Front Med (Lausanne). 2024 May 28;11:1382836. doi: 10.3389/fmed.2024.1382836 (PMC11165061; doi:10.3389/fmed.2024.1382836)
Supplement: Supplementary file 1 [file Data_Sheet_1.ZIP › data/GCST90274823_5e6/823.forest.pdf]

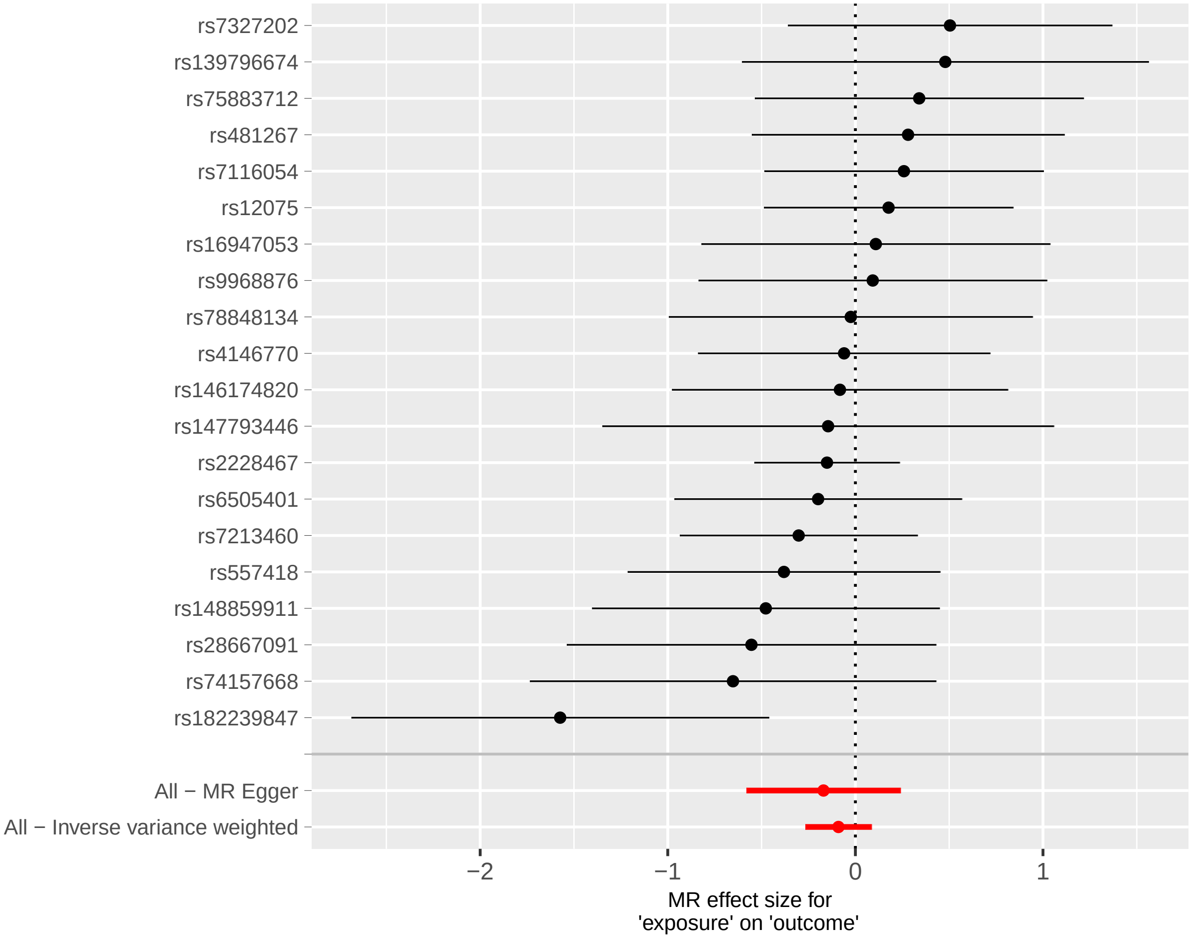
rs7327202
rs139796674
rs75883712
rs481267
rs7116054
rs12075
rs16947053
rs9968876
rs78848134
rs4146770
rs146174820
rs147793446
rs2228467
rs6505401
rs7213460
rs557418
rs148859911
rs28667091
rs74157668
rs182239847
All − MR Egger
All − Inverse variance weighted
−2
−1
0
1
MR effect size for
'exposure' on 'outcome'
